# Electrospun Poly(ethylene Terephthalate)/Silk Fibroin Composite for Filtration Application

**DOI:** 10.3390/polym13152499

**Published:** 2021-07-29

**Authors:** Alena Opálková Šišková, Katarína Mosnáčková, Jakub Hrůza, Jaroslava Frajová, Andrej Opálek, Mária Bučková, Katarína Kozics, Petra Peer, Anita Eckstein Andicsová

**Affiliations:** 1 Polymer Institute of Slovak Academy of Sciences, Dúbravská cesta 9, 845 41 Bratislava, Slovakia; katarina.mosnackova@savba.sk; 2 Institute of Materials and Machine Mechanics, Slovak Academy of Sciences, Dúbravská cesta 9, 845 13 Bratislava, Slovakia; andrej.opalek@savba.sk; 3 Advanced Technologies and Innovation, Institute for Nanomaterials, Technical University in Liberec, Studentská 1402/2, 461 17 Liberec, Czech Republic; jakubhruza1@seznam.cz; 4 Faculty of Arts and Architecture, Technical University in Liberec, Studentská 1402/2, 460 01 Liberec, Czech Republic; jaroslava.frajova@tul.cz; 5 Institute of Molecular Biology, Slovak Academy of Sciences, Dúbravská cesta 9, 845 51 Bratislava, Slovakia; maria.buckova@savba.sk; 6 Cancer Research Institute, Biomedical Research Center, Slovak Academy of Sciences, Dúbravská cesta 9, 845 05 Bratislava, Slovakia; katarina.kozics@savba.sk; 7 Institute of Hydrodynamics of the Czech Academy of Sciences, v. v. i., Pod Patankou 5, 166 12 Prague 6, Czech Republic; peer@ih.cas.cz

**Keywords:** poly(ethylene terephthalate), silk fibroin, electrospun membrane, air filtration, antibacterial activity, comfort properties

## Abstract

In this study, fibrous membranes from recycled-poly(ethylene terephthalate)/silk fibroin (r-PSF) were prepared by electrospinning for filtration applications. The effect of silk fibroin on morphology, fibers diameters, pores size, wettability, chemical structure, thermo-mechanical properties, filtration efficiency, filtration performance, and comfort properties such as air and water vapor permeability was investigated. The filtration efficiency (FE) and quality factor (Q_f_), which represents filtration performance, were calculated from penetration through the membranes using aerosol particles ranging from 120 nm to 2.46 μm. The fiber diameter influenced both FE and Q_f_. However, the basis weight of the membranes has an effect, especially on the FE. The prepared membranes were classified according to EN149, and the most effective was assigned to the class FFP1 and according to EN1822 to the class H13. The impact of silk fibroin on the air permeability was assessed. Furthermore, the antibacterial activity against bacteria *S. aureus* and *E. coli* and biocompatibility were evaluated. It is discussed that antibacterial activity depends not only on the type of used materials but also on fibrous membranes’ surface wettability. In vitro biocompatibility of the selected samples was studied, and it was proven to be of the non-cytotoxic effect of the keratinocytes (HaCaT) after 48 h of incubation.

## 1. Introduction

Following the current pandemic situation worldwide and prognosis published up to date by World Health Organization (WHO), there is no doubt that polymer fibrous membranes like filters or masks against COVID-19 are currently one of the most demanded products ever [1,2,3]. With this respect, every alternative and fabrication method should be seriously considered [4,5,6,7]. 

Electrospinning belongs among the most attractive methods for fabricating the fibrous membranes, which is a facile and effective approach for forming fine fibers in the micro- up to nanometers scale under the application of electric field [8]. Furthermore, this method offered the possibility to also prepare fibrous polymer products from synthetic or natural polymers or virgin and recycled polymers (plastic wastes) [9,10,11]. 

Electrospun products have already been studied for many applications such as wound healing [12,13], tissue engineering [14,15], drug-releasing and drug target delivery systems [16,17], sensors [18,19], membranes [20,21], batteries [22,23], solar cells [24,25], catalysts [26,27], protecting clothing [28,29], separation [30], decommissioning [31], and environmental remediation [32]. However, the randomly arranged ultrafine fibers in the electrospun membranes, the high surface area to volume ratios, nano-porosity, good mechanical properties, and vapor permeability of such membranes pre-destined them for filtration membranes and especially for personal protection as face masks against very fine dirt and bacteria, but also viruses with dimensions of about 100 nm [33,34,35,36]. 

Different polymeric materials have been used for producing electrospun membrane for filtration including cellulose acetate (CA) [37], silk fibroin (SF) [38], poly(lactic acid) (PLA) [39], polyamide-6 (PA6), poly(vinyl alcohol) (PVA) [40], polyvinylacetate (PVAc) [37], polyacrylonitrile (PAN) [41,42], polyvinylidene fluoride (PVDF) [43], or poly(ethylene terephthalate) (PET) [33,44]. 

It has already been published that the filtration efficiency of electrospun membranes could be high, even very close to 100%. However, the efficiency depends on the basis weight of the electrospun non-woven textile, the diameter of fibers in the membrane, and porosity [45]. Moreover, pressure drop and air or water vapor permeability are significant parameters for assessing filtration media properties relating to user comfort in personal protection applications [46]. Therefore, it is always challenging to increase efficiency while meanwhile maintaining low pressure drops. 

However, according to the latest statistics, PET accounts for 5% of the total annual production of plastics. It is up to 18.2 million tons, eventually ending up in landfills or incinerators [47]. Of this, only about 20% is used for recycling. Poly(ethylene terephthalate) is the standard thermoplastic polyester [48], which has excellent mechanical, thermal, and chemical properties and good dimensional stability. It is highly flexible, semi-crystalline, and shows excellent electrical insulating properties and high strength [49]. Poly(ethylene terephthalate) is the most common polymer for food and beverage packaging [50,51]; however, it can be seen in applications as automotive [49] or even medicine [52].

On the contrary, silk fibroin (SF) is obtained from natural silk cocoons type *Bombyx mori*. It is favorable due to its unique properties, including biocompatibility, biodegradability, minimal inflammatory reactions, and water vapor, as well as oxygen permeability [53,54]. The blending of silk fibroin, in combination with PET, is a novel approach that can use plastic waste and recover it for a new product with added value. By adding the silk, it is considered to give the better user comfort properties such as air and water vapor permeability to the composite membrane. Good air and water vapor permeability of electrospun silk fibroin mats has been already reported by many authors [55,56,57].

The main objective of the study is getting a membrane that will have good user comfort properties while mechanical properties are maintained.

In the present study, the electrospun membranes containing PET from bottle waste with varying contents of silk fibroin, according to the author’s best knowledge for the first time, were investigated. The composite membranes were characterized thoroughly. Scanning electron microscopy (SEM) was used for observing morphology, wettability of membranes was tested by measuring of water contact angle, the chemical composition was confirmed by attenuated total reflectance—Fourier transforms infrared spectrometry (ATR-FTIR), mechanical and thermal properties were investigated by dynamic mechanical thermal analysis (DMTA) and thermogravimetric analysis (TGA), respectively. This study’s main objective was to prepare the filtration medium with the ambition to serve as personal protection against fine dirt, bacteria, or even viruses with convenient user comfort properties. Therefore, the filtration efficiency, air, and water vapor permeability were tested. In the end, the antibacterial activity and biocompatibility were determined.

## 2. Materials and Methods

### 2.1. Materials

Commercially degummed silk fiber from *Bombyx mori* (Zhejiang, China) was used for our experiments. The municipal waste available beverages bottle was used as a source of poly(ethylene terephthalate) (r-PET) with the molar mass *M_w_* = 4.4 × 10^4^ g·mol^−1^ and molar-mass dispersity *Đ_M_* = 1.42. The molar mass measurement has already been described in our previous work [33]; 1,1,1,3,3,3-hexafluoro-2-propanol (HFIP, >99.0% purity) was purchased from TCI Tokyo Kasei (Tokyo, Japan). Dichloromethane p.a. (DCM, 99.8% purity) was purchased from Lach-Ner (Bratislava, Slovakia). Phosphate Buffer Saline (PBS) was purchased from Sigma-Aldrich (Weiheim, Germany) and absolute ethanol SOLVANAL, 99.8% from Centralchem (Bratislava, Slovakia). Di-ethylhexyl-sebacate (DEHS, >97.0% purity) was purchased from Palas GmbH (Karlsruhe, Germany). Casein-peptone lecithin polysorbate broth (CPLP broth) (base) was purchased from Merck, Burlington, MA, USA. Gram-positive bacteria *Staphylococcus aureus* (CCM 4223) and the Gram-negative bacteria *Escherichia coli* (CCM 3954) were purchased from the Czech Collection of Microorganisms, Masaryk University (Brno, Czech Republic). The human keratinocyte cell line HaCaT (T0020001) was purchased from AddexBio (San Diego, USA). Dulbecco’s Modified Eagle Medium (DMEM), fetal calf serum (FCS), and antibiotics (penicillin 100 U/mL; streptomycin 100 µg/mL) were purchased from Gibco BRL (Paisley, UK). 3-(4,5-Dimethyldiazol-2-yl)-2,5-diphenyltetrazolium bromide (MTT) was purchased from Calbiochem (Merck Millipore, Darmstadt, Germany).

### 2.2. Methods

#### 2.2.1. Preparation of Polymer Solutions for Electrospinning

(a) The r-PET solution was prepared in concentrations 10% (*m/V*) in the blend of 1,1,1,3,3,3-hexafluoro-2-propanol (HFIP) and dichloromethane (DCM). Pieces of post-consumer PET bottle were weighted into the vials, and the solvent HFIP was added into the vial. The solution was stirring intensively on magnetic plate EKA with an intensity of 750 rpm for 3 h. After dissolving in HFIP, the DCM was added to reach the required concentrations. The final solvent ratio in the solution was 30/70% *V/V* HFIP/DCM.

(b) A total of 2 g of silk fibers wer dissolved in 10 mL of a 9.3 M lithium bromide solution by stirring for 4 h at 60 °C to obtain a 20% (*m/V*) solution. The homogeneous solution was dialyzed for 2 days in distilled water using the dialysis membrane (12–14 kDa, Sigma-Aldrich, Saint Louis, MO, USA). The aggregates that occurred during dialysis were removed by centrifugation (10 min, 10,000 rpm, 25 °C). Obtained purified silk fibroin (SF) was subsequently lyophilized and kept in the freezer [58]. Finally, SF was dissolved in HFIP for electrospinning. The final concentration of the silk solution was 8% *m/V*.

The r-PET solution and silk solution were blended to obtain the solutions for electrospinning ratios, as summarized in Table 1.

#### 2.2.2. Electrospinning Process (ESP)

The fibrous mats were prepared using an electrospinning device under ambient temperature (23 ±1 °C, H = 57 ± 2%) in a horizontal spinning configuration with a flat-end needle with a 0.8 mm (21 gauge) inner diameter. The working distance was 12 cm. The applied voltage was between 16–18 kV, with positive polarity, and voltage was driven by a high-voltage power supply (Spellman SL-150W, Bochum, Germany). The solutions were fed by a single syringe pump model NE-1000 (New Era Pump Systems, Inc., Farmingdale, NY, USA). The feeding rate was 0.15 mL·h^−1^. The electrospun fibers were collected on aluminum foil.

#### 2.2.3. Morphology of Fibers and Pores Size

The morphology of investigated electrospun fibrous mats was observed by scanning electron microscopy (SEM) and JSM Jeol 6610 microscope (Jeol Ltd., Tokyo, Japan) at accelerated voltage 15 kV. The samples were sputtered with a thin layer of gold. Software AzTec (Springfield, NJ, USA) was used to collect figures and process the results. The average diameter of the fibers in the mats was measured utilizing Image J software (LOCI, University of Wisconsin, Madison, WI, USA). At least 100 segments were measured on the 5 independently prepared samples to ensure the accuracy of the average diameters of the fibers and their distributions.

The procedure described by Bandeira et al. [59] was used to prepare samples for SEM analysis of biofilms on electrospun fibers after antibacterial testing. First, the samples were washed twice with phosphate buffer solution (PBS, pH 7.4). Next, electrospun fibers were fixed with 4% paraformaldehyde for 30 min. Next, the samples were washed twice with PBS for 10 min and distilled water for 10 min. Subsequently, the samples were dehydrated with the addition of 25%, 50%, 70%, and 95% ethanol for 10 min and absolute ethanol twice for 15 min at room temperature.

The average pore size of membranes was determined from the SEM images with Adobe Creative Suite software (CS5, Adobe Systems Inc., San Jose, CA, USA) and calculated from more than 60 values on the 5 independently prepared samples.

#### 2.2.4. Attenuated Total Reflectance—Fourier Transform Infrared Spectrometry (ATR-FTIR)

Spectrophotometer Nicolet 8700 (Thermo Fisher Scientific, Madison, WI, USA) for Fourier transform infrared spectra recording, with DTGS TEC detector in mid-infrared scan range 600–4000 cm^−1^ with resolution 4 cm^−1^, was used in the absorbance mode. In addition, the spectrophotometer was equipped with a thermoelectrically cooled (TEC) fast-recovery DTGS detector. The spectra were measured in reflectance mode using the ATR (Attenuated Total Reflectance) accessory (Ge crystal was used as an optical window).

#### 2.2.5. Water Contact Angle (WCA)

Static water contact angle measurements of all investigated mats were performed at room temperature (22 ± 1°). Water droplets were used with a drop volume of 20 µL. The camera Canon Power Shot SX130 (Tokyo, Japan) was used for taking images. The baseline was estimated at the surface of the investigated mats and droplet interaction. The tangential line from the point of contact and the droplet’s outer surface was drawn using the ImageJ software (LOCI, University of Wisconsin, Madison, WI, USA). The angles between these two lines were recorded as the mean contact angle. The contact angle was assessed from at least 5 values on the 3 independently prepared samples.

#### 2.2.6. Mechanical Analysis

The tensile test was performed at room temperature using a Dynamometer Instron 4301 (Instron Corporation, Norwood, MA, USA) following standard ASTM D638. Seven testing strips for each formulation were cut from the electrospun mats with the dimensions of the tested strip area of 15 × 15 mm with a thickness of approximately 0.1 mm. The initial length of the tested strips was 120 mm because of better handling, and the gripping distance was 50 mm. A testing rate of 1 mm·min^−1^ was applied until 0.5% deformation was reached, and then the rates were increased to 20 mm·min^−1^. Average values of the tensile strength (σ_TS_), elongation at break (ε_B_), and Young’s modulus (*E*) was determined from the stress–strain curves. The mechanical analysis was assessed from at least 5 values on the 3 independently prepared samples.

#### 2.2.7. Dynamic Mechanical Thermal Analysis (DMTA)

Dynamic mechanical thermal analysis was performed using the Dynamic Mechanical Analyser DMAQ800 (TA Instruments, New Castle, DE, USA) within the temperature range from −20 °C to 160 °C with a heating rate of 3 °C·min^−1^. The measurements were carried out in tensile mode at 1 Hz frequency with deformation amplitude of 20 μm. The storage modulus (E´), loss modulus (E”), and loss tan delta (tan δ = E/E”) were determined for at least three specimens of each sample formulation. The mechanical analysis was assessed from at least 5 values on the 3 independently prepared samples.

#### 2.2.8. Thermogravimetric Analysis (TGA)

Linseis Combined thermal analyzer L75/L81/2000 (Linseis Messgeraete GmbH, Selb, Germany) was used for thermogravimetric measurements. Approximately 20 mg of the investigated samples were loosely filled into a smaller cylindrical crucible (height: 14.0 mm, diameter: 6 mm) of TG. The analyses were carried out in the nitrogen atmosphere with the flow 12 L.h^−1^. The temperature was increased from 30 °C up to 500 °C with the heating and cooling rate of 10 °C·min^−1^.

#### 2.2.9. Filtration Efficiency (FE) and Quality Factor (Q_f_)

The filtration efficiency of selected samples (r-PET, r-PSF3, r-PSF6, and SF) was determined precisely on instrument MFP 1000 HEPA (Palas GmbH, Karlsruhe, Germany) according to the requirements of the standard EN 1822 for high effective air filters (EPA, HEPA, and ULPA) and EN 149 for respiratory protective devices, filtering half masks to protect against particles. DEHS fluid suitable for producing steady aerosols was used as testing particles with the particles size 120–2460 nm. Face velocity 5.3 cm.s^−1^ and total volume flow was 32 L·min^−1^.

The quality factor (Q_f_) judges the relative overall performance of different membranes is calculated from the measurement of filtration efficiency (FE) and drop pressure (ΔP). It is defined as in Equation (1) [45].

(1) $$Q_{f}=\frac{-\ln\left(1-FE\right)}{\Delta P}$$

*Q_f_* is fairly independent of basis weight [60].

The filtration efficiency was assessed from the 3 independently prepared samples.

#### 2.2.10. Air Permeability (B)

FX3300 air permeability tester III (Artec Testnology, Hertogenbosch, Netherlands) was used to measure air permeability. The measurement pressure was set to 100 Pa, and the test sample’s dimension was 20 × 20 cm. The results were evaluated according to EN ISO 9237. The air permeability was assessed from the 3 independently prepared samples.

#### 2.2.11. Water Vapor Permeability (WVP)

The vapor permeability was measured using the PERMETEST Sensora Skin Model (Sensora, Liberec, Czech Republic) [61,62]. The device provides measurements required in the ISO Standard 11092. The measurements were carried out at laboratory temperature 20–22 °C, and the laboratory water vapor concentration (humidity) of the parallel airflow 45–60% was applied. The samples with dimensions 12 × 12 cm were used. The water vapor permeability was assessed from the 3 independently prepared samples.

#### 2.2.12. Antibacterial Activity (R)

Antimicrobial activity of electrospun pure r-PET, r-PSF6, and SF pure fibrous mats was determined with adherence to the procedure following ISO 22196:2011 for *S. aureus* and *E. coli* [63]. Bacterial suspensions were prepared at concentration between 2.5 x 10^5^ to 10 × 10^5^ cells·mL^−1^. 400 μL of the suspension was put on the surface of specimen 40 mm × 40 mm in each sample, covered with a square piece of polyethylene film. The samples and the polyethylene films were placed under UV light for 30 min to sterilize them just before the experiments. After the contact time of 24 h, the specimens (samples) were rinsed with CPLP broth (10 mL; Casein-peptone lecithin polysorbate broth (base)) on a petri dish, and the value for CFU·mL^−1^ was determined. Log reduction in the number of living and viable cells of tested bacteria (R) was calculated according to Equation (2):

(2) $$R=\left(U_{t}-U_{0}\right)-\left(A_{t}-U_{0}\right)=U_{t}-A_{t}$$

where *U*_0_ is the average value for the common logarithm of the number of viable bacteria, in cells·cm^−2^, recovered from the control samples (r-PET) immediately after inoculation, *U_t_* is the mean for the common logarithm of the number of viable bacteria, in cells·cm^−2^, recovered from the control samples after 24 h, *A_t_* mean for the common logarithm of the number of viable bacteria in cells·cm^−2^ is retrieved from the test sample (r-PSF6) after 24 h.

Antimicrobial test results are given as means of 3 experiments ± SD. The differences between the given groups were tested for statistical significance using Student’s t-test (* *p* < 0.05; ** *p* < 0.01; *** *p* < 0.001).

#### 2.2.13. Biocompatibility

The cells (HaCaT) were cultivated in Dulbecco’s Modified Eagle Medium (DMEM) supplemented with 10% fetal calf serum (FCS) and antibiotics (penicillin 100 U·mL^−1^; streptomycin 100 µg·mL^−1^). The cells were cultured in a humidified atmosphere of 5% CO_2_ at 37 °C.

Cytotoxicity of selected samples r-PET, r-PSF6, and SF were determined using the MTT method. Briefly, 2 × 10^4^ cells were seeded in 96-well plates and cultured in a complete DMEM medium. The studied r-PET, r-PSF6, and SF were then added, and the cells were incubated at 37 °C in a 5% CO_2_ atmosphere for 24 and 48 h. Next, the samples were washed with phosphate-buffered saline (PBS) at the indicated time point, followed by incubation with 1 mg·mL^−1^ of MTT for 4 h. Then, the MTT was removed, and the formazan crystals were dissolved with dimethyl sulfoxide for 30 min. Absorbance at a wavelength of 540 nm was measured using an xMark microplate Spectrophotometer (Bio-Rad Laboratories, Inc., Hercules, CA, USA), and background absorbance at 690 nm was subtracted. The results are presented as mean ± SD in quadruplicates (*n* = 4) from three independent experiments.

## 3. Results and Discussion

In this study, post-consumer bottle PET and silk fibroin regenerated from the silkworm cocoons were selected as readily available and cheap (only a few cents per kilogram [64,65]) materials to prepare fibrous mat’s potential for filtration application. Polyethylene terephthalate is insoluble in common organic or aqueous solvents. The solvents in this study had to be selected regarding electrospinnability. PET has been already electrospun from a trifluoroacetic acid (TFA), a mixture of TFA/DCM [44,66,67] in various portions or from HFIP and HFIP/DCM [33,68]. In the case of silk fibroin, it has been proven many times that the SF could be electrospun from aqueous solutions only with the aid of an auxiliary polymer [69,70]; however, it was successfully electrospun from formic acid (FA), TFA, and HFIP [58,70,71].

Regarding the solubility of both parts of the composite in the HFIP and the previous good results of authors, it has been used in the mixture with DCM [33]. HFIP has been used as a solvent of low-soluble synthetic polymers as well as protein-based natural polymers [72,73]. Dichloromethane is widely used in the pharmaceutical industry as a process solvent for that the Food and Drug Administration (FAD) has established residue tolerances [74]. The solution mixtures prepared according to Table 1 were processed by electrospinning process according to the parameters mentioned in Section 2.2.2.

The quality of fibrous mats is affected by several parameters [75,76]. Therefore, the processing parameters such as applied voltage, flow rate, needle top to collector distance, and concentration of solutions were selected by gradually experimenting to get the beads-free fibers with the average diameter to ensure high filtration efficiency. This effort was made because the morphology of the fibers was shown to be highly correlated to the membrane properties, particularly in terms of fiber diameter and porosity [60]. In addition, the suitable concentrations of individual PET and SF stock solutions and parameters, such as flow rate, working distance, and voltage, were adjusted in preliminary experiments.

### 3.1. Morphology of Electrospun Mats and Average Diameter of the Fibers

**Figure 1:**
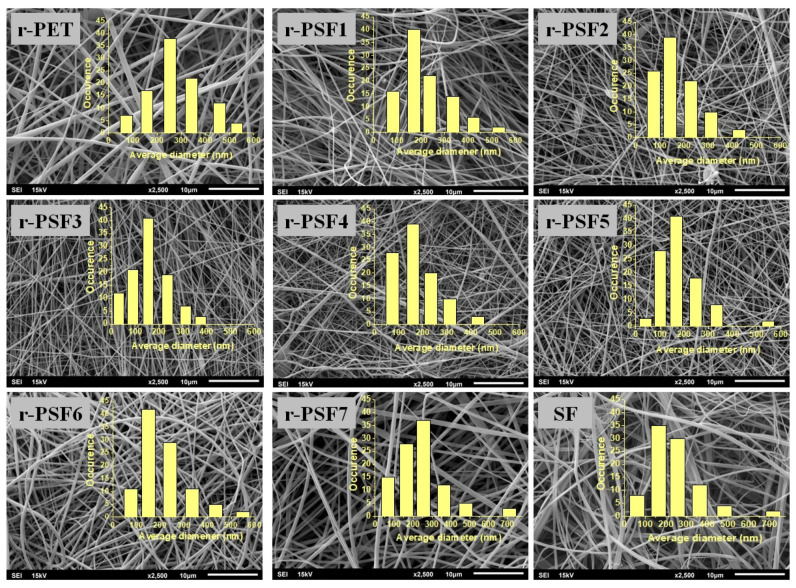
SEM micrographs of electrospun mats with varying amounts of silk.

Free-standing fibrous mats were produced. The morphology was assessed by SEM, and the micrographs of all prepared membranes are displayed in Figure 1. The micrographs show that the membranes contain randomly oriented, continuous, smooth fibers and beads were rarely observed. Compared to the pure r-PET nanofiber, it was found that the addition of silk fibroin induced the formation of thinner nanofiber.

Measuring the diameters of nanofibers is one of the important tools to assess the uniformity of mats [77]. Therefore, mean averages of fiber diameters were calculated from 100 individual fibers for each sample. Summarized average fiber diameters in investigated membranes are listed in Table 2. The average diameter was changing with the increasing concentration of silk fibroin. As the content of SF increased, the total polymer solution concentration decreased, and the critical concentration was achieved for sample r-PSF3 with an average diameter of 127 ± 50 nm. This can be caused by the reduced viscosity of the blended solution because a lower viscosity of the solution leads to a thinner fiber diameter [78]. Then with the further increase of the SF concentration, the average diameter grew again, and fiber diameters distribution increased. Generally, with the further decreasing total concentration, the formation of the beads was expected. However, the results here show that the nanofiber characteristics cannot be attributed to a single composition parameter. Thus, the interaction effects of the formulation compositions must also be taken into account, as it has already been studied [79].

As can be seen from Table 2, the estimated pore size of r-PET and SF mats was larger than that of fibrous composites (except r-PSF7) and showed a decreasing trend with the decrease of fiber diameter. This trend is consistent with other electrospun nanofiber membrane investigations, where the larger pore size is obtained when fiber diameter increased [43].

### 3.2. Water Contact Angle (WCA)

The WCA of pure r-PET, SF, and composite r-PSFs membranes were investigated. The values of contact angles are given in Table 2. The r-PET fibrous membrane was more hydrophobic, with a mean water contact angle of 95 ± 3°. However, the water droplet spread out almost immediately in pure SF case and penetrated the fibrous membrane. The contact angle of SF was 10 ± 1 °C at the moment of drop impact. Next, in fibrous composites r-PSF3, r-PSF4, r-PSF5, and r-PSF6, the hydrophobicity decreased with the increased proportion of SF, indicating that SF improved the hydrophilicity in comparison to r-PET. These results demonstrate that the SF considerably affects the surface wettability of the fibrous membranes, making the structure more hydrophilic, which is in good agreement with available literature [80]. Silk fibroin fibers can be hydrophilic due to the high number of the amino group and carboxylic acid domains they contain [81]. In liquid/aerosol filtration applications, the filtration performance is closely related to the wettability of the filtration media due to different shapes of droplets, barrel, or clamshell, on the fiber surface and various positions of liquid films present on the filter surface during filtration processes [82]. Furthermore, wettability is an advantageous property that can save operating costs in filtration due to the need for lower energy to push the liquid during filtration through the hydrophilic filtration medium [83].

### 3.3. ATR-FTIR Analysis of Investigated Fibrous Mats

**Figure 2:**
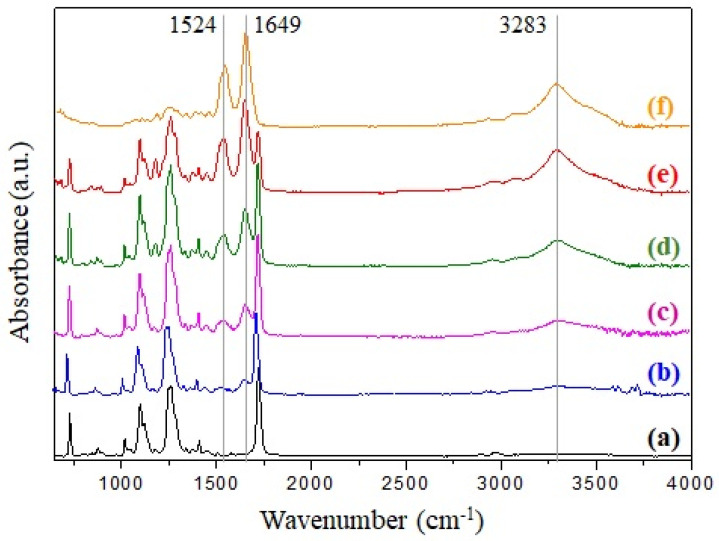
FTIR spectra of (a) r-PET, (b) r-PSF4, (c) r-PSF5, (d) r-PSF6, (e) r-PSF7, (f) SF.

ATR-FTIR is a common tool for investigating the chemical compositions and molecular conformations of polymers and composite materials [84]. The ATR-FTIR spectra of the electrospun r-PET, SF, and r-PSF4–r-PSF7 with SF concentration from 28.5–70.5% (*V/V*) are shown in Figure 2.

The common characteristics bands of poly(ethylene terephthalate) are observed in Figure 2a. The band at 724 cm^−1^ is attributed to the interaction of polar ester groups and benzene rings, at 1265 cm^−1^ to the terephthalate group (OOCC_6_H_4_–COO), at 1050 cm^−1^ to ester C–O bond, and band at 1714 cm^−1^ is corresponding to C=O stretching [85,86].

Enhancing of the bands in the case of r-PSF4, r-PSF5, r-PSF6, r-PSF7 (Figure 2b–e) belonging for the amide groups of silk at 1240 cm^−1^ (amide III, C–N stretching), 1649 cm^−1^ (amide I, C=O stretching), 1524 cm^−1^, and 3283 cm^−1^ (amide II, N–H bending) correspond to the increasing of SF concentration, which was attributed to the random coil, α-helix, and β-sheet structure of silk fibroin [58,87,88]. These bands are compared to the bands of pure silk fibroin located in the spectrum depicted in Figure 2f. Electrospun mats with a lower concentration of SF than 28.5% (*V/V*) were investigated by ATR-FTIR as well; however, the typical bands at 1524 cm^−1^ and 1649 cm^−1^ have not been observed yet, and therefore the spectra are not shown here. The ATR-FTIR measurements of prepared membranes were carried out from both sides of the membranes; however, the results are compared and therefore are not presented.

### 3.4. Mechanical Properties

**Figure 3:**
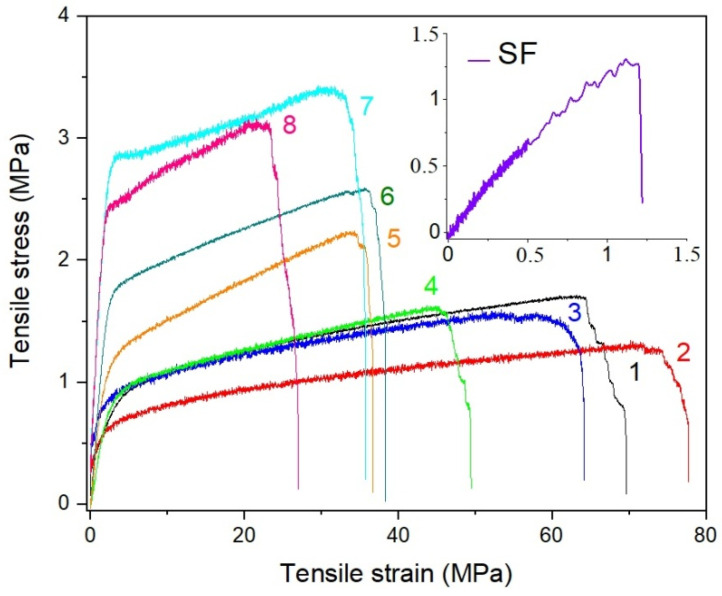
Stress–strain curves for r-PET electrospun mats contained (1) r-PET pure, (2) r-PSF1, (3) r-PSF2, (4) r-PSF3, (5) r-PSF4, (6) r-PSF5, (7) r-PSF6, (8) r-PSF7. The inset shows the stress–strain curve of crude electrospun SF.

The mechanical properties of the electrospun membranes intended to serve as filtration media in personal protection are important parameters because such membranes are stretched in all directions during the application. To investigate the characteristics of electrospun r-PSFs mats, tensile tests for samples with a basic weight 10–12 g·m^−2^ were performed to understand how the different compositions affected mechanical properties. The stress–strain curves for pure r-PET and all r-PSFs membranes differ in SF concentration for direct comparison are shown in Figure 3. There are significant differences in terms of their mechanical behavior. As can be seen from the inset of Figure 3, the pure SF shows brittle behavior, reflecting weak mechanical resistance. The determination of the mechanical properties of the fibrous mats revealed that the increasing SF loading was leading to σ_TS_ increases due to SF fibrous structure, reflected in high tensile strength and flexibility.

On the contrary, the elongation at the break slightly increased with a small drop in tensile strength typically observed after the addition of plasticizer due to higher free volume. Increasing the SF volume, the r-PSF mats became more brittle with a continuous decline in elongation at break (ε_B_/σ_TS_) as a function of SF content. Addition of the highest SF volume (r-PSF7) lead to extensive deterioration of mechanical properties, resulting in twice lower elongation at break than the pure r-PET mat (ε_B_/σ_TS_ values are summarized in Table 3).

**Figure 4:**
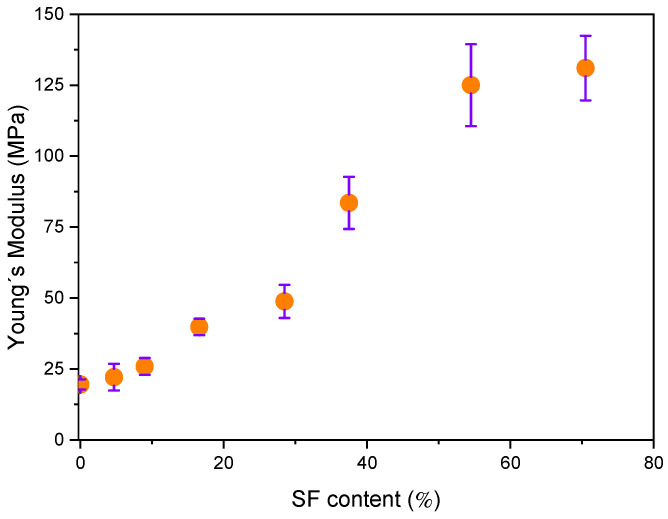
Changes of Young´s modulus for r-PET electrospun mats as a function of content SF was varying from 0 to 70.5 *V/V* % (from r-PET to r-PSF7).

Young’s modulus (E) of the electrospun pure r-PET, r-PSF mats, containing different SF amounts is shown in Figure 4. Young´s modulus is attributed to the strength of the interaction between fibers within the material that does not significantly depend on its thickness [89]. It was observed that increasing SF content in r-PSF samples increases the E. Similar behavior was reported by Gobin et al. [90], which attributed these changes to more numerous interactions of SF, with presented chitosan in the form of strong molecular hydrogen bonding. Hydrogen interactions can occur not only at the polymer filler interfacial, but also in the form of mutual particle interactions, especially at higher fibroin concentrations resulting in significant increase in stiffness.

### 3.5. Dynamic Mechanical Thermal Analysis

**Figure 5:**
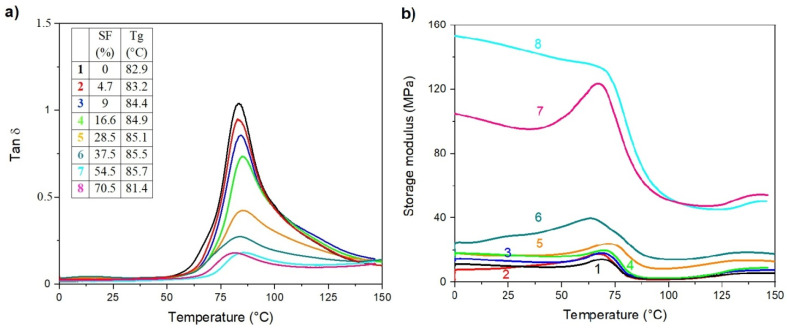
Evolution of loss factor (tan δ) (**a**) and storage modulus (**b**) with temperature of electrospun membranes containing (1) r-PET, (2) r-PSF1, (3) r-PSF2, (4) r-PSF3, (5) r-PSF4, (6) r-PSF5, (7) r-PSF6, (8) r-PSF7.

The DMTA is a sensitive characterization method giving additional information at the level of phase or molecular structure within the polymer, such as crosslinking, branching, and crystallinity. The behavior of loss factor (tan δ) and storage modulus (E´) for the electrospun r-PET mats contained varying SF concentrations and are compared in Figure 5. The pure r-PET exhibits one peak assigned to glass transition temperature (T_g_) at approximately 83 °C. Silk fibroin affected T_g_ slightly shifted to the lower temperatures and reduced the maximum loss factor peak with increasing the SF content. Apparently, the presence of fibrous structure can significantly affect the mobility of the polymer chains due to acting as a heavy barrier for relaxation processes required for followed arrangement of the polymer chains. Figure 5b shows the dependence of E´ as a function of temperature for all the investigated electrospun mats. Generally, the observed changes in E´ are associated with interface adhesion between polymer and fillers which can be related to changes within a material structure such as crosslinking, aging, or degradation [91]. As is clearly shown in Figure 5b (black line), the E´ of neat r-PET mat shows a progressive decrease at around 80 °C due to the glass transition from the glassy to rubbery state. The addition of SF (from 4.7 to 70.5%) results in a gradual increase in E´ at 25 °C as a consequence of limited chain mobility, reflecting in pronounced stiffness of the material. For the low SF concentrations, there is negligible increase in E´ of r-PET/SF electrospun mats compared to neat r-PET while at higher SF concentration (over 28.5%), and the E´ increases dramatically due to effective reinforcing of presented SF and reaching approximately 15 times higher value compared to neat r-PET.

### 3.6. Thermogravimetric Analysis (TGA)

**Figure 6:**
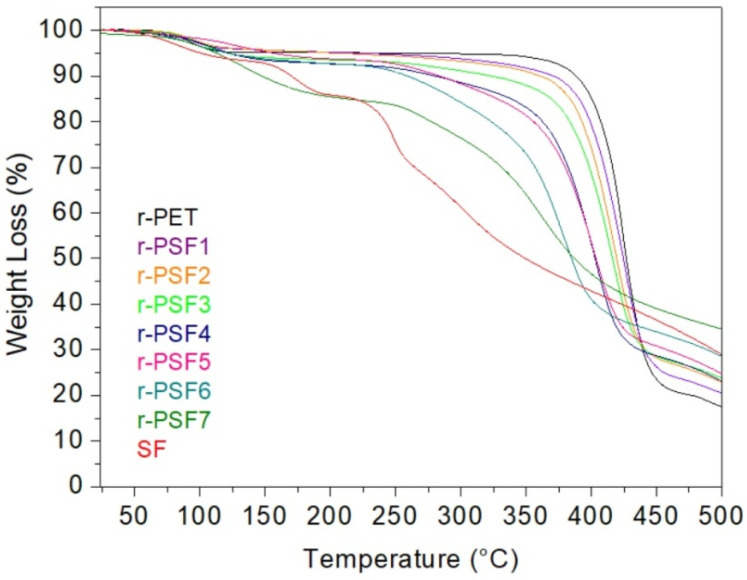
Thermogravimetric analysis of investigated membranes at temperatures between 30 and 500 °C.

The TGA was used to estimate the thermal stability of the investigated samples. The results are shown in Figure 6. The first weight loss is observed close to 100 °C due to moisture loss and residual solvents. This first weight loss is around 5% and agrees in all samples.

The TGA curve of the electrospun r-PET shows stability up to the decomposition temperature at around 400 °C. After that, the degradation reaction ceased at a residue accumulation of 20%. It is in good agreement with published results when thermal degradation occurs under an inert environment [92]. Subsequently, the decomposition temperature decreases with the increasing concentration of SF in the investigated sample; however, the accumulation of residue is increasing. In r-PSF6, the decomposition temperature is around 250 °C, and the accumulation of residue at 500 °C is 30%. Unlike the r-PET, the sample r-PSF7 exhibits two steps of weight loss. First is at 100 °C, and this decreasing passes into the plane between 180–250 °C. The weight loss is 15%. Then, the second and most significant weight loss starts at 250 °C, corresponds to the degradation of silk. The accumulation weight loss at 500 °C is 35%. The TGA curve of r-PSF7 exhibits the similarities of both types of polymers, r-PET, and SF as well. In the TGA curve of SF, the decomposition is also in the two steps; however, the plane after the first weight loss is shorter, between 180–230 °C, as it was published by [93,94].

### 3.7. Filtration Efficiency and Comfort Properties

Advanced filtration technologies need to be designed to provide effective and reliable capture of particles under 300 nm sizes. Electrospun fibers have been widely used in many applications, especially in air filtration, due to the significantly large specific area, small fiber diameter, and porosity.

**Figure 7:**
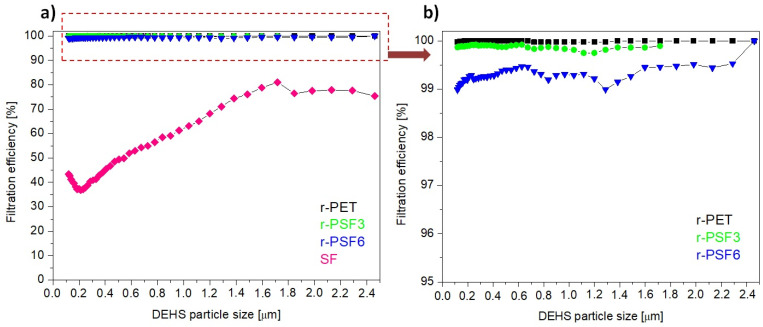
Filtration efficiency of investigated samples with basis weights between 10–12 g·m^−2^; (**a**) r-PET (black sign), r-PSF3 (green sign), r-PSF6 (blue sign), SF (magenta sign); (**b**) detail of the curves at high efficiency.

**Figure 8:**
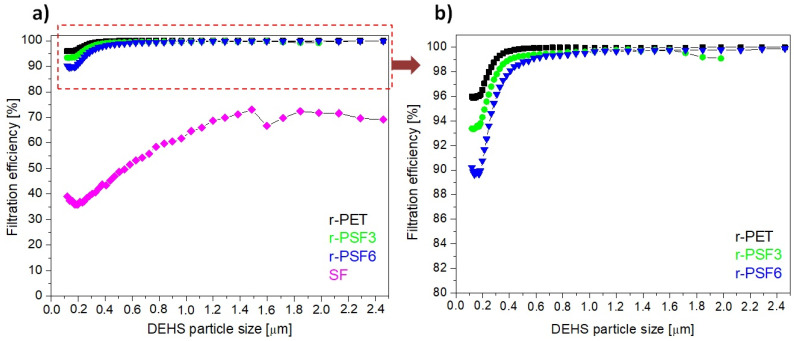
Filtration efficiency of investigated samples with basis weights between 1.75–3.23 g·m^−2^; (**a**) r-PET (black sign), r-PSF3 (green sign), r-PSF6 (blue sign), SF (magenta sign); (**b**) detail of the curves at high efficiency.

Herein, the filtration efficiency was established for the selected electrospun samples r-PET, r-PSF3, r-PSF6, and SF. The r-PSF3 was selected due to the smallest fiber diameter (Table 2) that indicates the high filtration efficiency and r-PSF6 for the equal amount of r-PET and SF in the mixture. The membranes were electrospun in two different basis weight ranges: 10–12 g·m^−2^ (Table 4, Figure 7) and 1.75–3.23 g·m^−2^ (Table 6, Figure 8).

Figure 7a shows the most representative curves of filtration efficiency of each type of the investigated membranes. Figure 7b shows the detail of the curves representing the higher FE of the three discussed membranes. Figures show the trend of filtration efficiency in dependence on the DEHS particle size. The trend is not linear, and this nonlinear behavior could be explained by the filtration performance governed by filtration mechanisms, including inertial impaction, interception, electrostatic attraction, diffusion, and gravity sedimentation [45,95,96]. The mechanisms can also participate in the filtration simultaneously.

It is shown that at basis weight in the range 10–12 g·m^−2^, the r-PET, r-PSF3 exhibited filtration efficiency of more than 99% and r-PSF6 of 98.99% filtration efficiency as listed in Table 4.

However, the pressure drop measured in all three samples was too high to classify the membranes according to EN149 (Table 5) and to apply these membranes for personal protection. The higher pressures drop the worse breathability. It is always challenging to increase efficiency while maintaining low pressure drops [45].

Nevertheless, the pressure drop of r-PET, r-PSF3, and r-PSF6 is adequate for applying to EPA (Efficient Particulate Air filter) and HEPA (High Efficiency Particulate Air filter) filters in air ventilation and air conditioning, and these membranes could be classified according to standard EN1822 (Table 5) [96]. In general, the lower the pressure drop, the lower the operating costs. The SF membranes exhibited just 43%, which is too low to be classified according to the mentioned standards. It follows that the FE depends not only on basis weight but also on the type of polymer and its properties [37]. The measured (exact basis weight, FE, and pressure drop) and calculated data of quality factor (*Q_f_*) are listed in Table 4. The trade-off parameter *Q_f_* evaluates the filtration performance of a given filtration medium. From Table 4 it can be observed that the highest value of *Q_f_* obtains sample r-PSF3 with the thinnest fiber diameter.

Figure 8a presents the most representative curves of filtration efficiency of each type of investigated membrane r-PET, r-PSF3, r-PSF6, and SF in the range of basis weight 1.75–3.23 g·m^−2^. Figure 8b shows the detail of the curves representing the higher FE of the discussed three membranes with higher filtration effectivity. The r-PET, r-PSF3, and r-PSF6 exhibited filtration efficiency of more than 90%. On the other hand, the SF membranes showed just 39% of FE. The measured (exact basis weight, FE, and pressure drop) and calculated data (*Q_f_*) are listed in Table 6.

The filtration effectivity, as well as the pressure drop, depends on the basis weight. On the other hand, the quality factor does not depend on the basis weight, and it is the biggest for the r-PSF6. The larger Q_f_ indicates a more excellent filtration performance, but not for very efficient filters because the efficiency growth over 90% is usually lower than the pressure drop growth. Therefore, the higher effectivity in r-PSF3 is more important; this can prove that the fiber diameter plays a role in the FE.

The range of application is determined by the type of filter FFP1, FFP2, and FFP3, which determines the amount and kind of trapped particles (Table 5). According to the FE in this series of the samples (Table 6), the r-PSF6 could be classified as FFP1, which corresponds to the standard face masks most commonly used by residents during the pandemic. The membranes r-PET, r-PSF3, and r-PSF6 could be classified again according to EN1822 to the class E10 and E11, as is listed in Table 6.

### 3.8. Air and Water Vapor Permeability

For the applications for which the membranes under investigation are intended, the air permeability (B) is a key indicator, because good air permeability provides good micro-environment. Moreover, better air permeability means better flux [98]. Therefore, there is a growing urge to investigate membrane air permeability. Herein, air permeability of the selected membranes r-PET, r-PSF3, r-PSF6, and SF was measured, and the results are listed in Table 7.

Many parameters, such as fiber diameter, pores size, basis weight, and the surface wettability of the membrane, can affect the air and water vapor permeability of nanofibers membrane. In this study, air permeability was measured for the selected samples with a higher basis weight (10–12 g·m^−2^) and the smaller basis weight (1.75–3.23 g·m^−2^). The dependence of air permeability on the basis weight is evident from Table 7. With an increasing basis weight of membrane, the air permeability decreased, which is in good agreement with the literature regardless of the type of polymer [28]. Comparing the results with WCA from Table 2 shows increasing air permeability with decreasing the WCA. However, the dependence on the fiber diameter has not been confirmed in this study. This parameter seems to vary from case to case [99,100]. The dependence of air permeability on the amount of SF in the membrane is evident from Table 7. The higher the concentration of SF, the better the air permeability.

Water vapor permeability (WVP) is a fundamental parameter in evaluating the comfort characteristics of membranes. The WVP in protecting clothing represents the ability of perspiration transfer what is also the case of filtration masks [101]. The results of water vapor permeability for the samples with a higher basis weight (10–12 g·m^−2^) was slightly higher than in the smaller basis weight (1.75–3.23 g·m^−2^) (Table 7). The WVP decreased in the range of 1–6%. The most significant 6% decrease was recorded for r-PET, which was the fibrous mat with the largest basis weight and at the same time with the largest fiber diameters. It cannot be said that with increasing fiber diameter the WVP decreases, because permeability was measured and compared for the samples prepared in the same conditions and with similar structural characteristics (similar fibers diameter) from each basis weight and samples with different compositions. However, according to the available literature, WVP is only slightly dependent on fiber diameters, but the effect of basis weight has already been observed [102]. The dependence of WVP on the SF amount in the membrane is not evident. The changes are negligible, especially in the case of membranes with lower basis weight.

The expectations that the air and water vapor permeability was confirmed only in the case of air permeability. The higher amount of SF in the investigated membrane, the better air permeability.

### 3.9. Antibacterial Activity

Many authors described the antibacterial activity of silk fibroin. The published results, as well as opinions on this topic, seem clear. Silk is not considered a polymer with antibacterial activity [89,103]. On the other side, there have been published studies that have shown that the antibacterial activity of polymers in different forms is influenced by many factors related to its preparation, processing [104], possibly mixing with other polymers [50,105] or active molecules, drugs, and biocides [106,107,108].

In this study, the antibacterial activity of the electrospun r-PET, SF, and r-PSF6 was tested without any inhibiting agent by contact method, following the ISO22196:2011 against *S. aureus* as gram-positive and *E. coli* as gram-negative bacteria. In addition, the bacterial viability of both used strains was investigated after contact of 24 h. Results are listed in Table 8.

The numbers of recovered viable bacteria were established. The result of the antibacterial activity testing is the following already published studies [103].

**Figure 9:**
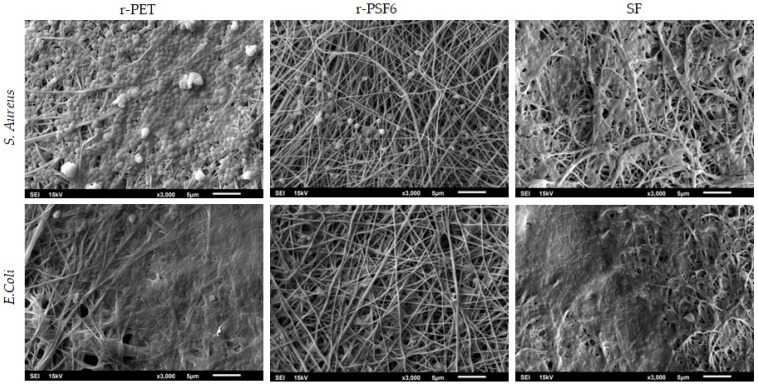
SEM micrographs of r-PET and r-PSF6 and SF show antibacterial efficiency against *S. Aureus* and *E. Coli*.

Pure SF and r-PET are not antibacterial, and the bacterial films were forming on the membrane surface confirmed by SEM micrographs (Figure 9). Unlike published studies mentioned above and all the expectations, the experiment results showed that bacterial growth on the r-PSF6 was reduced for both tested strains. The viability of *E.coli* and *S. aureus* reduction for more than 94% was observed compared to r-PET and SF. The antimicrobial activity was calculated as R = 1.3 for *S. aureus* and *E. coli*. The explanation of this result could lie in the critical surface characteristics that are desirable for reducing bacterial binding and that are related to surface energy, roughness, and wettability [109]. It was shown by Yean et al. that the electrospun polystyrene surface with a WCA of 95° gave the highest level of bacterial (*E. coli*) adhesion [110]. This corresponds with earlier research showing that a surface with WCA in the range 54–130° had higher bacterial adhesion by promoting hydrophobic interaction between bacterial membrane and a solid surface [111]. In the case of this study, the antibacterial activity of the r-PSF6 could be explained by the higher wettability and lower WCA 43 ± 4°.

### 3.10. Biocompatibility

**Figure 10:**
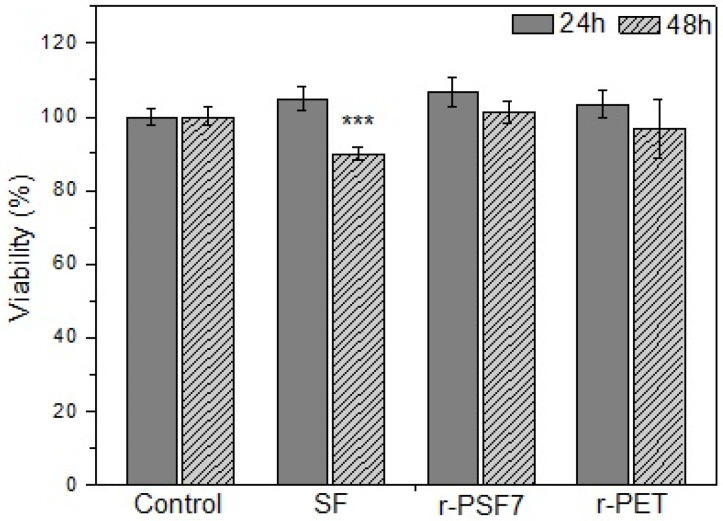
Biocompatibility of SF, r-PSF6, and r-PET mats after 24 h and 48 h incubation, respectively. Cell viability was determined by MTT assay. The results are presented as mean ± SD of three independent experiments. The statistical significance is shown by asterisks (*** *p* < 0.001).

The biocompatibility of SF in many forms has already been studied by many researchers [53,112,113]. The majority of the amino acids in silk fibroin are glycine (45.9%) and alanine (30.3%), which show minimal chemical reactivity and give the SF unique features including biocompatibility, biodegradability, and host–implantation integration. Silk fibroin has been due to its biocompatibility studied in many applications such as tissue and nervous system regeneration [114,115], wound dressing [58,116], drug release systems [117], and sutures [118]. Herein, the cytotoxic effects of 24 and 48 h exposure of the investigated samples were evaluated in HaCaT cells by the MTT assay. HaCaT cells are the immortalized human keratinocytes, so the primary type of cells found in the outermost layer of the skin. In humans, they form 90% of epidermal skin cells [119,120]. They were selected due to the intended close skin contact with investigated materials. The results are summarized in Figure 10. As expected, the SF mats used in this study have proven to be biocompatible with the HaCaT cells used after 24 and 48 h incubation.

As shown, r-PET and r-PSF6 had no cytotoxic effect on the HaCaT cells. Their viability slightly increased after 24h of incubation, while after 48h it decreased back to the control level. These results agree with the literature, which shows excellent biostability and biocompatibility of a Food and Drug Administration (FDA)-approved polymer PET [121].

## 4. Conclusions

Fibrous free-standing membranes were fabricated from the blend of mechanically stable recycled PET and silk fibroin extracted from the cocoon’s silkworm *Bombyx Mori.* The membranes were prepared by electrospinning. Up until now, the membranes from the mixture of r-PET and SF were not published; therefore, the membranes were characterized by elemental techniques to investigate the basic features and subsequently were tested as aerosol filtration membranes to assess the suitability of these membranes for filtration. The mechanical, thermal, and chemical properties are affected by both present types of polymers.

The wettability of the r-PSFs composite membranes is modified distinctively with the increasing SF amount. The filtration efficiency (FE) and quality factor (Q_f_), which represents filtration performance, were calculated from penetration through the membranes using DEHS aerosol particles ranging from 120 nm to 2.46 μm. The basis weight of the membranes influenced FE and Q_f_. Studied membranes with basis weight in the range of 10–12 g·m^−2^ exhibit FE 43–99% and Q_f_ 0.012–0.024 Pa^−1^ and membranes with basis weight in the range 1.7–3.2 g·m^−2^ exhibit FE 39–96% and Q_f_ 0.018–0.039 Pa^−1^. The membrane r-PSF6 with the FE 90.23% was classified as FFP1 type according to the standard EN149. These results show the eventuality to use the investigated membrane r-PSF6 for personal protection. The other tested membranes showed too high a pressure drop. The effectivity of tested membranes r-PET, r-PSF3, and r-PSF6 were classified into the class E11, E10, and E10, in the case of lower basis weight and into the class H13, E12, and E12 in the case of higher basis weight according to EN1822. Antibacterial activity of r-PET, r-PSF6, and SF electrospun membranes has been tested. The air permeability was improving with the higher amount of SF in the composite. On the other hand, the addition of SF has no impact on the water vapor permeability. The viability of two strains of bacteria, *S. aureus* and *E. coli*, was reduced by around 95% after 24 h of contact time. The biocompatibility of investigated samples SF, r-PSF6, and r-PET have been proven. The studied samples were shown to have a non-cytotoxic effect after 48 h of incubation compared to control. Given the results presented, it can be concluded that the material would also be suitable for use in filtration application against bacteria, viruses, or other particles, even in nanoscale.

## Figures and Tables

**Table 1:** Content of solutions prepared for electrospinning.

Sample	10% (m/V) of r-PET [mL]	8% (m/V) of SF [mL]	SF Content in % (*V/V*)	Total Concentration (%)
r-PET	4	0	0	10
r-PSF1	4	0.2	4.7	9.9
r-PSF2	4	0.4	9	9.8
r-PSF3	4	0.8	16.6	9.6
r-PSF4	4	1.6	28.5	9.4
r-PSF5	4	2.4	37.5	9.2
r-PSF6	4	4.8	54.5	8.9
r-PSF7	4	9.6	70.5	8.5
SF	0	4	100	8

**Table 2:** List of average diameters (AD), the mean pore size of the fibers in the mats, and contact angles of all investigated electrospun membranes.

Sample	AD ± SD (nm)	Mean Pore Size ± SD (nm)	Contact Angle (°)
r-PET	232 ± 118	850 ± 331	95 ± 3
r-PSF1	168 ± 83	654 ± 344	94 ± 2
r-PSF2	134 ± 63	352 ± 124	92 ± 3
r-PSF3	127 ± 50	348 ± 121	86 ± 1
r-PSF4	139 ± 76	398 ± 104	61 ± 4
r-PSF5	146 ± 103	427 ± 140	51 ± 9
r-PSF6	149 ± 89	480 ± 188	43 ± 4
r-PSF7	253 ± 125	975 ± 345	31 ± 5
SF	202 ± 128	828 ± 380	10 ± 1

**Table 3:** Summarized results of mechanical testing.

Sample	Mechanical Analysis	
	σ_TS_ ± S _σ_ [MPa]	ε_B_ ± S _ε_ [%]
r-PET	1.62 ± 0.11	64.90 ± 14.8
r-PSF1	1.28 ± 0.19	67.30 ± 16.2
r-PSF2	1.32 ± 0.21	59.50 ± 6.82
r-PSF3	1.49 ± 0.13	40.50 ± 8.10
r-PSF4	1.85 ± 0.26	31.00 ± 6.68
r-PSF5	2.75 ± 0.29	36.80 ± 8.10
r-PSF6	3.45 ± 0.50	33.50 ± 4.30
r-PSF7	3.10 ± 0.37	25.80 ± 4.60
SF	1.35 ± 0.34	1.89 ± 0.60

**Table 4:** List of results from the filtration activity of investigated fibrous mats: SF pure, r-PSF3, r-PSF6, and r-PET pure. The basis weights of the investigated mats were between 10–12 g·m^−2^. The membranes were classified according to Standard EN149 and EN1822. The values are listed in the form mean ± SD (standard deviation).

Sample	Basis Weight (g·m^−2^)	Thickness (mm)	* E_MPPS_ (%)	ΔP (Pa)	Q_f_ (Pa^−1^)	Filter Class According to EN149	Filter Class According to EN1822
**r-PET**	12.01±0.01	0.11 ± 0.002	99.97 ± 0.2	414 ± 21	0.019 ± 0.001	High ΔP	H13
**r-PSF3**	11.15 ± 0.01	0.11 ± 0.002	99.85 ± 0.4	272 ± 14	0.024 ± 0.001	High ΔP	E12
**r-PSF6**	10.25 ± 0.01	0.10 ± 0.001	98.99 ± 1.9	315 ± 15	0.015 ± 0.001	High ΔP	E11
**SF**	11.00 ± 0.02	0.11 ± 0.002	43.34 ± 6.4	47 ± 8	0.012 ± 0.002	No classification	No classification

*E_MPPS_—efficiency of most penetrated particles.

**Table 5:** Division of respiratory protection into classification according to standard EN149 and EN1822, minimum efficiency of safety, maximal acceptable pressure drop at the total volume flow 30 L·min^−1^, and examples of protection [96,97].

**Filtration Class According to the EN149**	**Minimal Efficiency of MPPA (%)**	**Recommended** **Pressure Drop (Pa) (at 30 L·min^−1^)**	**Protection**
FFP1	≥80	60	Solid inert particles, aerosols without particular toxicity, e.g., calcium carbonate, plaster, brick dust, pollen, and fur.
FFP 2	≥94	70	Biological and carcinogenic compounds, harmful solid particles, toxic or irritating aqueous aerosols, e.g., silica, sodium carbonate, iron, wood and glass dust, water-soluble pesticides, grain, mold, fungi, exhaust gasses.
FFP3	≥99	100	Biological compounds, toxic solids, aqueous aerosols, e.g., TBC bacteria, asbestos, radioactive beryllium particles.
**Filtration Class According to the EN1822**	**Minimal Efficiency of MPPA (%)**	**Recommended** **Final Pressure Drop (Pa)**	**Protection**
E10	≥85	250–1000 *	germs, bacteria, metallic-oxide smoke
E11	≥95		
E12	≥99.5		viruses, tobacco smoke, soot
H13	≥99.95		oil fumes, radioactive suspended particulates
H14	≥99.995		aerosols

* Commercially available filters in classified into the class according to the EN1822.

**Table 6:** Summarized results from measurement of the filtration activity of investigated fibrous mats: SF, r-PSF3, r-PSF6, and r-PET. The basis weights of the investigated mats were between 1.75–3.23 g·m^−2^. The membranes were classified according to Standard EN 149. The values are listed in the form mean ± SD (standard deviation).

Sample	Basis Weight (g·m^−2^)	Thickness (mm) (μm)	*E_MPPS_ (%)	ΔP (Pa)	Q_f_ (Pa^−1^)	Filter Class According to EN149	Filter Class According to EN1822
**r-PET**	3.23 ± 0.02	0.08 ± 0.003	95.98 ± 0.2	123 ± 4.4	0.026 ± 0.001	High ΔP	E11
**r-PSF3**	2.08 ± 0.02	0.07 ± 0.001	93.38 ± 0.9	92 ± 2.6	0.030 ± 0.001	High ΔP	E10
**r-PSF6**	2.65 ± 0.01	0.08 ± 0.001	90.23 ± 2.7	59 ± 2.2	0.039 ± 0.001	FFP1	E10
**SF**	1.75 ± 0.03	0.07 ± 0.001	39.01 ± 6.4	27 ± 9.6	0.018 ± 0.002	No classification	No classification

*E_MPPS_—efficiency of most penetrated particles.

**Table 7:** Results of air permeability (B) and water vapor permeability (WVP). The values are listed in the form mean ± SD (standard deviation).

Sample	Basis Weight	1.75–3.23 (g·m^−2^)		10–12 (g·m^−2^)	
		B (L·mm·s^−1^)	WVP (%)	B (L·mm·s^−1^)	WVP (%)
**r-PET**		53.4 ± 2.7	89.2 ± 1.5	39.8 ± 2.4	94.7 ± 1.0
**r-PSF3**		117.0 ± 4.2	86.5 ± 1.7	54.6 ± 3.9	89.2 ± 1.4
**r-PSF6**		149.9 ± 5.7	88.9 ± 1.7	85.0 ± 7.0	89.9 ± 1.3
**SF**		201.3 ± 8.3	89.3 ± 2.0	150.0 ± 9.1	89.5 ± 1.9

**Table 8:** Antibacterial activity of the investigated electrospun mats. The values are listed in the form mean ± SD.

Tested Microorganism	Sample	The Number of Bacteria Recovered at 24 h Contact Time (CFU·cm^−2^)	Log of the Number of Bacteria Recovered at 24 h Contact Time (CFU·cm^−2^)	Antimicrobial Activity (R)	Reduction (%)
***S. aureus*** **CCM 3953**	r-PET	390,000 ± 18,000	5.59 ± 0.26	-	-
	r-PSF6	21,000 ± 1200	4.26 ± 0.24	1.3 ± 0.02	94.62 ± 6.6
	SF	350,000 ± 16,400	5.54 ± 0.26	-	-
***E. coli*** **CCM 3988**	r-PET	550,000 ± 25,000	5.74 ± 0.26	-	-
	r-PSF6	30,000 ± 1600	4.48 ± 0.24	1.3 ± 0.02	94.55 ± 6.4
	SF	480,000 ± 21,300	5.68 ± 0.25	-	-
